# Two-Level Blockchain System for Digital Crime Evidence Management

**DOI:** 10.3390/s21093051

**Published:** 2021-04-27

**Authors:** Donghyo Kim, Sun-Young Ihm, Yunsik Son

**Affiliations:** 1 Department of Computer Science and Engineering, Dongguk University, Seoul 04620, Korea; donghyo@dgu.ac.kr; 2 Department of Computer Engineering, Pai Chai University, Daejeon 35345, Korea; sunnyihm@pcu.ac.kr

**Keywords:** blockchain, crime evidence management, digital forensic, smart contract

## Abstract

Digital evidence, such as evidence from CCTV and event data recorders, is highly valuable in criminal investigations, and is used as definitive evidence in trials. However, there are risks when digital evidence obtained during the investigation of a case is managed through a physical hard disk drive until it is submitted to the court. Previous studies have focused on the integrated management of digital evidence in a centralized system, but if a centralized system server is attacked, major operations and investigation information may be leaked. Therefore, there is a need to reliably manage digital evidence and investigation information using blockchain technology in a distributed system environment. However, when large amounts of data—such as evidence videos—are stored in a blockchain, the data that must be processed only within one block before being created increase, causing performance degradation. Therefore, we propose a two-level blockchain system that separates digital evidence into hot and cold blockchains. In the criminal investigation process, information that frequently changes is stored in the hot blockchain, and unchanging data such as videos are stored in the cold blockchain. To evaluate the system, we measured the storage and inquiry processing performance of digital crime evidence videos according to the different capacities in the two-level blockchain system.

## 1. Introduction

Recently, digital evidence has been used as definitive evidence in criminal investigations and trials. For instance, in 1985, a murder occurred at a rest stop in Oklahoma, USA, but a strong suspect could not be found. However, DNA from cigarette butts smoked by the murderer at that time was stored until 35 years later, when the criminal could be charged [1]. Moreover, in South Korea, a criminal was arrested for a crime that was committed in 1988 after the evidence of sexual assault and murder cases that remained unsolved after 18 years was compared with DNA that had been permanently stored [2]. Thus, it is becoming important to store and manage digital evidence for a long time so that it is not tampered with or damaged [3].

Table 1 shows the amount of digital evidence stored in Korea as of 2020. As the use of devices such as smartphones, CCTV, and event data recorders (EDRs; also known as black boxes) has expanded recently, the amount of digital evidence used in criminal investigations continues to increase. This means that the amount of digital evidence that needs to be stored and managed has increased, and standard and systematic management is required. However, there is currently no standard model for digital evidence storage in Korea or abroad, so this management is not effective [3]. In particular, South Korea generally uses a hard disk, which is a physical storage device, to store digital evidence collected by investigators. This hard disk drive is used from the collection of the digital evidence in the early stages of a criminal investigation until it is submitted to the court. In this digital evidence management system, there is potential for the damage and manipulation of digital evidence during the criminal investigation process, because the chain of custody cannot be maintained. Therefore, such evidence has not been used in court, because its reliability cannot be guaranteed [4]. To solve this problem, [5] proposed a system for storing and managing digital evidence in a reliable and transparent blockchain, wherein the digital evidence is encrypted and stored in the blockchain in the form of a hash, and all blocks refer to the hash of the previous block. In addition, because all of the nodes participating in the blockchain are connected like a chain in order to share digital evidence, it has the advantage of ensuring the transparency of digital evidence.

However, because CCTV and EDRs have been proven to provide important evidence in recent criminal investigations, the necessity of safely managing digital crime evidence videos is also increasingly clear [6]. When large amounts of data, such as crime evidence videos, are stored in the blockchain, the amount of data that must be processed within one block increases when the transaction is confirmed and the block is created. Therefore, performance degrades in the blockchain system [7]. In this paper, to address this problem, we propose a two-level blockchain system consisting of hot and cold blockchains to efficiently manage the large amount of crime data considering the characteristics of criminal evidence. The criminal evidence is characterized by its use as evidence to ensure immutability. Blockchain systems for managing large amounts of data have been widely studied, such as hierarchical, multi-level, and on/off-chain blockchains. However, the existing hierarchical blockchain or multi-level blockchain is suitable for effectively managing the data of multiple terminals or nodes, such as IoT devices [8,9]. Moreover, there are some studies that combine on-chain and off-chain scaling. The on-chain consists of blockchains, but the off-chain is composed of external data, not blockchains, and so there is an oracle problem that requires verification every time [10]—that is, there has been no research on an efficient blockchain system that considers data usage. Thus, in this paper we divide the blockchain into two levels for retrieval and utilization, while maintaining security based on the Hyperledger Fabric framework—one of the consortium blockchains. Crime evidence data can be categorized into two types as frequently modified data: investigative information; and data for recording, such as videos. This system separates and stores crime evidence into hot evidence—which has been highly modified in each criminal investigation (e.g., investigation or identity information)—and cold evidence, which has not been modified (e.g., criminal evidence videos). 

The rest of this paper is organized as follows: Section 2 explains the Korean criminal investigation process for digital evidence management, and describes prior research on digital crime evidence video management. In addition, we introduce the basic concept of a blockchain, the Hyperledger Fabric framework for constructing blockchain systems, and techniques for storing digital crime evidence videos in a blockchain. Section 3 proposes the design and implementation of the two-level blockchain system, consisting of hot and cold blockchains for crime evidence video management. Section 4 shows performance experiments on the proposed two-level blockchain system, and Section 5 concludes the study and presents future work.

## 2. Related Work

### 2.1. Digital Crime Investigation

Digital forensics deals with legal evidence left in computers and digital recording media. This is a procedure and method for legally identifying and demonstrating the facts of a specific activity that occurred using a digital device. Digital forensics tools are used daily by inspectors and analysts in local, state, and federal law enforcement agencies. More and more organizations encounter data that cannot be analyzed with today’s tools, because of format incompatibility, encryption, or a lack of education [11]. Currently, several studies are being conducted to improve operational efficiency through a systematic approach to expressing forensic data and performing forensic operations. The US federal court system is exploring ways to improve the accountability of digital surveillance. The authors of [12] illustrate how accountability and secrecy are simultaneously achievable when modern cryptography is brought to bear. To do so, the authors implement a hierarchical form of multi-party computation that reflects the hierarchy of the court system. 

Currently, the criminal investigation process in Korea for digital evidence management consists of seven steps: initial investigation, internal investigation, arrest, completion of investigation, request for analysis, transmission of digital evidence and investigation documents, and trial [3]. The first step, the initial investigation, is carried out when a crime occurs. This is initiated by on-site investigation agencies that recognize the characteristics of a case. In the second step, the internal investigation, criminal evidence is collected by visiting the site after a warrant has been issued for a promising suspect. The suspect is arrested in the third step, the arrest process. In the fourth step, the investigation is concluded, and on-site investigators obtain photos of the criminal evidence. In the fifth step, the criminal evidence for the case is collected, and information about the investigator and investigation are registered in the Korea Information System of Criminal Justice Services (KICS). If a detailed analysis is required, a request is sent to the project analysis team of each local police agency, and the results are returned. In the sixth step, the hard disk that contains the original criminal evidence, investigation documents, and criminal evidence analysis results is sent to the prosecution and recorded in the investigation documents for storage. In the last step, digital evidence that has been sent to the prosecutor’s office and reviewed is submitted to the court for use as evidence in the trial.

### 2.2. Digital Evidence Management Research in Korea

**Figure 1:**
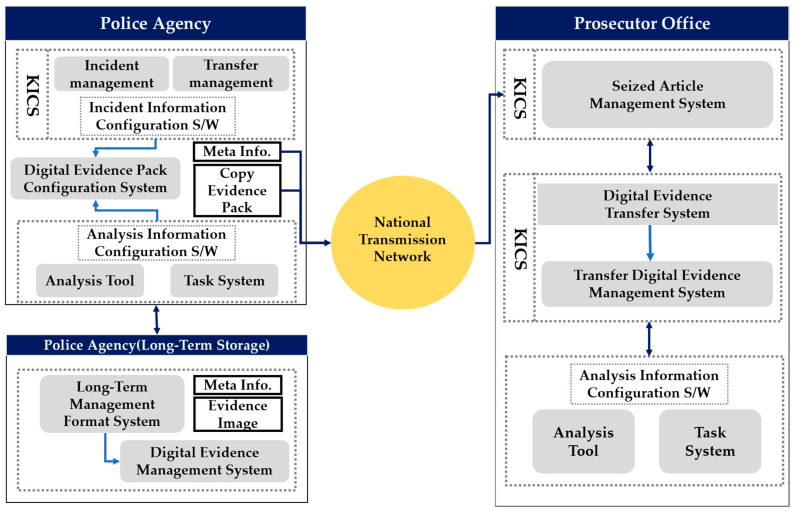
System structure of the existing integrated digital evidence management system.

Studies on ways of improving the original identity, integrity, and reliability of criminal digital evidence obtained in the investigation process in Korea have been conducted [4,5]. However, most of these studies focus on identifying the characteristics of digital data, and establishing a life-cycle-based management environment with integrated digital evidence management. Figure 1 shows one proposed integrated digital crime management system in Korea. Its aim was to integrate digital evidence and investigation information through the national transmission network by linking KICS—a criminal justice information system operated by the police, prosecutors, and courts—and the case management systems of the national police agency [4].

However, in previously proposed integrated digital evidence management systems in Korea, a server was operated as a centralized system in an integrated server/client environment. A centralized system is a system that processes all data from each region on a central computer. In other words, it refers to a system in which one specific computer processes data, instead of using all the computers in a server system connected through a communication network to process data. A centralized system has the advantages of easily integrated data management, and low management costs, because the data are concentrated on a central server. However, in such a system, when a server is attacked, important operations and investigation information of the relevant institution may be leaked, and the continuity of storage cannot be maintained for digital evidence. Therefore, digital evidence and investigation information must be managed reliably and transparently in a distributed environment, not in the existing centralized digital integrated management system.

### 2.3. Blockchain-Based Large Data Management 

With the recent developments in the field of big data, many studies have investigated the storage and management of large amounts of data, such as videos and images, in blockchains in various fields, such as medicine, forensics, and education [13,14,15,16,17,18,19]. A permissioned blockchain framework for forensic applications of connected vehicles was proposed in [20]. This framework manages the collected vehicle-related data, with the ability to access a variety of information about the cars. LEChain [21] was proposed to manage lawful evidence flow and all of the court data based on blockchains. LEChain uses a random signature that anonymously authenticates the identity of witnesses, in order to manage the overall flow of evidence and all court data during evidence collection and police investigations, and to protect the privacy of witnesses.

However, a blockchain is basically a structure in which all nodes record the shared data on the network. In other words, each node has a repository that stores data, called a distributed ledger, which is placed on multiple nodes. When there is a written request from the user, it broadcasts the status to all nodes so that each node stores the same state. A transaction in a blockchain means that a certain state and data are written onto the blockchain. Currently, one of the methods of sharing the same state for each system, as well as making contracts and agreements, is blockchain technology. However, in the blockchain, even if a number of physical computers are connected, the calculations are concentrated on a single node, and the scalability of block capacity becomes a problem [22,23]. 

To address this problem of blockchain scalability, research on a hierarchical blockchain model has been studied [8]. Each level of the hierarchical blockchain maintains multiple local blockchains, records local transaction activity, and allows partial views of different subsets of blockchain records to be maintained on the next level of the blockchain. However, the blockchain architecture cannot be scaled enough to meet the demands of large-scale data traffic. This is because we need to find a balance between coping with high throughput and handling a large number of nodes [9]. The existing hierarchical blockchain systems [8,9] are suitable for managing data that consist of multiple terminals or nodes, such as IoT devices. However, they cannot support the data with large files, such as crime evidence videos [24,25].

Research also has recently been attempted on on-chain scaling, which records data within the blockchain, and off-chain techniques, which record data outside the blockchain [26,27]. On-chain scaling approaches the scalability problem by expanding the block size of the blockchain, and is also called the big block method. As the size of the block increases, the fee within the block chain decreases, and a large amount of data can be recorded. However, as the capacity of the block increases, the block generation time increases. This is because data need to be processed only within one block when a transaction is shared within the blockchain and a block is created. Therefore, using big blocks degrades the performance of the blockchain system. Off-chain scaling is a method of storing data outside of the blockchain, and its main purpose is to reduce the data input to the block by utilizing an external network and block storage, without increasing the size of the block itself. In other words, it manages data by storing large amounts of data in external storage, and storing the reference values of the data, access authority information, and key values in the blockchain. There are existing studies [10,28] that combine on-chain and off-chain scaling; however, there is an oracle problem when importing external data into the blockchain [29].

Currently, most methods of storing and managing large amounts of data in the blockchain do so using the IPFS (InterPlanetary File System), which is an off-chain technique. The IPFS is a distributed file system designed to improve the speed of the request/response method, which is a problem with traditional HTTP Web communication. The IPFS allows large data files to be exchanged using peer-to-peer communication on nodes without centralized servers. Unlike HTTP Web, the IPFS can maintain a system with small nodes whether the system is compromised or not. Moreover, because it uses a file system based on the Merkle DAG [30], its file duplication and search abilities are fast. However, if blockchains and the IPFS are combined, system performance degrades because of the high network bandwidth needed for communication between the blockchains and the IPFS.

### 2.4. Blockchain and Hyperledger Fabric

A blockchain is a decentralized system in which each distributed computer handles tasks and exchanges its content or results, in contrast to focusing on a centralized computer to process all data as in a centralized system [31]. In other words, a blockchain has the advantage of being able to manage the same transactions transparently and reliably, by connecting them to the most recently generated blocks from the beginning of the chain and sharing them with all network nodes. Because of these advantages, the industry uses a variety of open-source frameworks for blockchain integration and application. Blockchains can be largely divided into public and private blockchains, also known as consortium blockchains, based on the type of participation. A public blockchain is an open blockchain that has no limits on nodes and is decentralized, where transactions are approved by consensus. These are highly reliable, because all participants can generate transactions, participate in notarization, and go through the mutual verification of all participants. In addition, since the transaction details are open to all participants, all nodes participating in the network must verify and approve them. However, due to the nature of this consensus system, it is difficult to expand the network, the processing speed is slow, and it is very difficult to change the rules once they have been established. A consortium blockchain is a semi-centralized blockchain, which is a blockchain that secures reliability and expands by connecting heterogeneous blocks generated in different blockchains by combining different private blockchains [32]. Such a network can be used only by a specific participating group. The manager is a participant in the consortium, and can change the rules according to the consensus of the participants. Since only authorized users can access the data, identification is possible, network expansion is easy, and transaction speed is fast compared to public blockchains. The permissioned blockchain provides an additional level of security by maintaining a layer of access control, so that only certain identifiable participants can perform certain tasks. It also maintains the identity of each blockchain participant in the network. Blockchains of this kind can selectively set restrictions during network configuration, and control the activities of various participants in the desired role [33].

**Figure 2:**
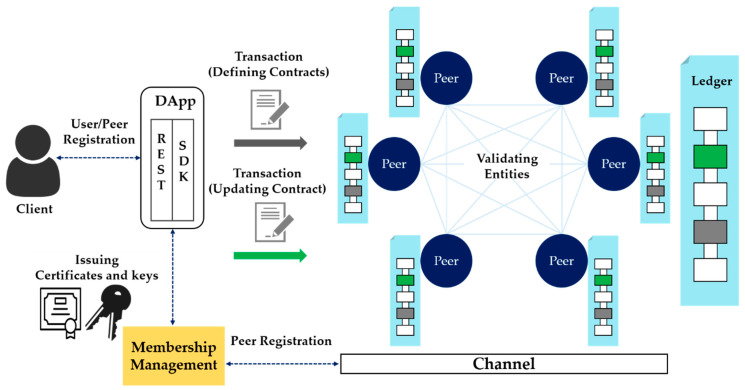
Hyperledger Fabric framework.

Hyperledger Fabric, one of the consortium blockchains, is a Linux Foundation Hyperledger project. It is an open-source framework for building blockchain infrastructure for business-to-business and business-to-consumer transactions [34]. Unlike Bitcoin or Ethereum, the representative open blockchain frameworks, Hyperledger Fabric is a consortium blockchain in which several organizations form a consortium and only authorized institutions can participate. As shown in Figure 2, a public key infrastructure (PKI)-based digital certificate, public key, and private key pair are issued by the authentication server, so that only users who have the authority to participate in the blockchain have access. As a result, only users with access rights to the channels constituting the independent environment can access the blockchain, which provides an independent transaction and block-sharing environment among participants. In addition, it guarantees the integrity of the data of the blockchain participants, and can share data and blocks within the blockchain. This means that a channel can be constructed and shared only among the participants who wish to share digital evidence and investigative information. 

Hyperledger Fabric consists of a blockchain network, a certificate authority (CA) server, and a client, as shown in Figure 2. In addition, membership information about peers and an orderer’s authority in the blockchain can be registered on the certification authority server. This system can be maintained by generating and distributing digital public keys, private keys, and other keys. A peer is a node in the network in Hyperledger Fabric that manages distributed ledgers and smart contracts, and various nodes exist depending on their roles. An orderer is a node that creates a real block, and the process of deploying blocks to all nodes is called consensus. Because the task is separated between the peer and the orderer, the load on the peer to execute and verify the transaction can be reduced, and parallel processing to perform various tasks simultaneously is possible. To create a block, transactions can be exchanged between each peer through a smart contract module in the blockchain, composed of source code. A smart contract is a type of contract that can be automatically signed and have its contents modified without an intermediary. In Hyperledger Fabric, the source code that executes the smart contract is called the chaincode, and it is installed on the peer to execute the transaction. Here, when a blockchain transaction occurs, authentication is received through a key issued by the CA server. CA is Hyperledger Fabric’s certification authority, which connects to ID registration and user registry LDAP, and has functions of issuing, renewing, and revoking registration certificates (ECerts) [35]. In the method proposed in this paper, the components of Hyperledger Fabric are utilized in order to store and manage crime evidence videos effectively.

## 3. Two-Level Blockchain System for Digital Crime Evidence Management

### 3.1. Design of the Two-Level Blockchain System

**Figure 3:**
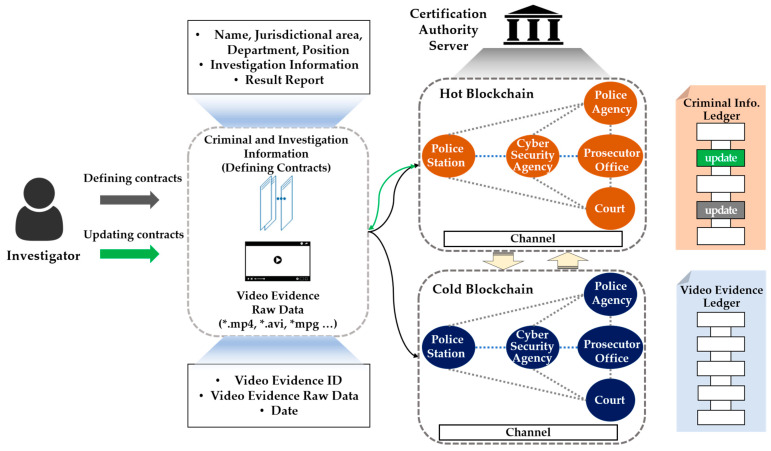
Two-level blockchain system.

Our proposed two-level blockchain system for efficient crime evidence management consists of two layers, managed by hot and cold blockchains (Figure 3). In the hot blockchain, investigation and identity information with frequent transaction fluctuations throughout the criminal investigation process is stored, and the cold blockchain stores digital crime evidence videos that do not require modification after storage.

Institutions corresponding to the national police agency, local police agency, cyber analysis team, prosecutor’s office, and courts—which are the peers in the channel—would form a consortium in order to participate in blockchain channels and share the identity and investigation information in the hot blockchain using the process shown in Table 2. When registering an identity, the authentication server issues a private key belonging to the national police agency peer to the on-site investigator. In Algorithm 1, the on-site investigator obtains the authority to access the hot blockchain through the issued private key, and can store the investigation and identity information. Users who do not have keys belonging to the national police agency peer cannot access or store data on a hot blockchain. The transactions delivered by the on-site investigator to the hot blockchain include ID, name, social security number, department, jurisdiction, date, investigation information, and evidence ID. In addition, only investigators with verified identities and those in the judicial system can transfer transactions to the chaincode. After the transaction has been delivered, a block containing the identity and investigation information is created through the chaincode, which is a predefined smart contract source code, and the transaction and block are distributed to all organizations participating in the blockchain.
**Algorithm 1** Save and Search of Hot & Cold Blockchain**Input:** Hot_Tx // Investigation and identity transaction    Cold_Tx // Evidence video and identity transaction    K // User Private key **output:** Save Success or Failure     Search Result of Hot_Tx and Cold_Tx01 Hot_ledger [] ← NULL // Hot Blockchain Ledger 02 Cold_ledger [] ← NULL // Cold Blockchain Ledger 03 D [] ← List // User Digital Certificate 04 **function** Save(Hot_Tx, Cold_Tx, K) 05   I ← 0 // User index 06   V ← 0 // Valid flag 07   F ← 2 // Full flag 08   while D[I] do 09    if D[I] ∋ K && !is Empty(HoT_Tx) 10      V ← V + 1 11      Append(Hot_ledger [I], HoT_Tx) 12    end if 13    if D[I] ∋ K && !isEmpty(Cold_Tx) 14      V ← V + 1 15      Append(Cold_ledger[I], Cold_Tx) 16    end if 17    I ← I + 1 18    end while 19   while true do 20    if V = F return true 21    else return false 22    end if 23   end while 24 **end function** 25 **function** Search(HoT_Tx.*_ID_*_and RNN,_ K) 26   I ← 0 // User index 27   R1 ← 0 // Hot_Tx 28   R2 ← 0 // Cold_Tx 29   while D[I] do 30    if D[I] ∋ K 31      if Hot_Tx[I].*_ID and RNN_*_=_ Hot_Ledger[i].*_ID_*_and RNN_ 32       R1 ← Hot_Ledger[I] 33      end if 34      if R1. *_EVI_ID_* = Cold_Ledger[I].*_EVI_ID_* 35       R2 ← Cold _Ledger[I] 36      end if 37     I ← I + 1 38   end while 39   return R1, R2 40 **end function**

To manage digital crime evidence videos, we can access the cold blockchain using the key used to verify the identity of the police agency peer in the hot blockchain. After identity verification, the on-site investigator obtains the right to create and distribute blocks by storing the original digital crime evidence video in the cold blockchain. Then, frequent transactions during the investigation process can access the hot blockchain in order to request the identity and investigation information, as well as accessing the cold blockchain through the evidence ID in order to retrieve evidence. In addition, it is impossible to modify a digital crime evidence video that is not allowed to be modified. In this way, the data stored in the hot and cold blockchains are created in blocks and cannot be modified or deleted, and they are shared with all peers in the blockchain system, thus enhancing transparency and reliability.

### 3.2. Implementation

**Figure 4:**
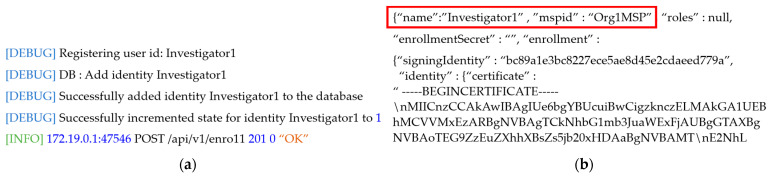
Identity registration by (**a**) institution, and (**b**) digital authentication issuance of the two-level blockchain system.

In the system proposed in this paper, we use Hyperledger Fabric, a blockchain construction framework, and Docker, a software virtualization framework, to implement the two-level blockchain. When managing a blockchain, a number of nodes are connected within the communication network in order to exchange data, so physical computers for each configured peer are required. To do this, a number of peers, authentication servers, orderers, and blockchain state databases are created as containers using Docker, and the blockchain infrastructure is maintained by mounting ledgers, blocks, transactions, and other components to the built container. In the two-level blockchain system, PKI-based encryption keys are issued and used as credentials, so that peers corresponding to the local police agency, cyber analysis team, prosecutor’s office, police agency, and courts can form a consortium and participate in the same channel. To enable the on-site investigator to store investigation and identity information and digital crime evidence videos in the hot and cold blockchains in the two-level blockchain system, the identity is registered in containerized peers and orderers. To this end, containerized peers participate in the channel to form a consortium, and an identity registration request is made to the authentication server using a PKI-based administrator digital certificate and private key. Figure 4 shows the result of requesting the authentication server to register an identity for each affiliated organization, and obtaining access to the two-level blockchain system in order to register the user identity to the peer of the hot and cold blockchain. We can confirm that the identities of the peers have been registered. Based on the registered identity shown in Figure 4, the authentication server issues the digital certificate and private key for each institution, and these are used for identity verification when accessing the hot and cold blockchains.

**Figure 5:**
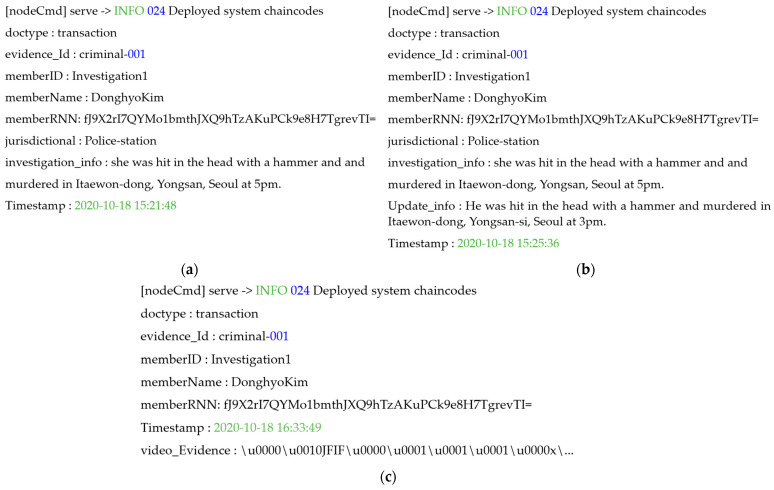
Save, update, and retrieve of the two-level blockchain system. (**a**) Saving the information of investigation and identity, (**b**) updating the information of investigation and identity, and (**c**) retrieving digital crime evidence videos.

Subsequently, the verified on-site investigator can access the two-level blockchain and obtain the authority to store investigation and identity information, as well as crime evidence videos. The on-site investigator delivers the data to the peer of the national police agency to which he or she belongs, in order to store investigation and identity information and video clips of criminal evidence in the blockchain. At this time, the investigation and identity information are transferred to the hot blockchain, and the crime evidence video is transferred to the cold blockchain chaincode factor to execute the contract. Figure 5 shows the result of the on-site investigator delivering the investigation and identity information to the hot blockchain, executing the chaincode, and finally requesting and updating the transactions stored in the ledger and state database. In the transaction, investigation and identification information such as ID, name, social security number, department, jurisdiction, date, investigation information, and evidence ID are stored in the ledger. Storage in the chaincode of the investigation and identity information is identified by the registrant’s ID, digital certificate, and private key. When a field investigator requests a transaction, the chaincode is executed and registered in the ledger of the national police agency peer.

**Figure 6:**
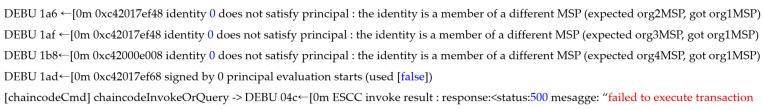
Failure to access the two-level blockchain system using an unregistered identity.

The cold blockchain, which is a core part of the system proposed in this paper, is accessed using the same digital certificate and key used when requesting identity registration for the hot blockchain. Subsequently, the on-site investigator stores the crime evidence video by passing the digital crime evidence video and part of the identity information stored in the hot blockchain as chaincode factors of the cold blockchain. In the chaincode of the cold blockchain, the crime evidence video delivered by the on-site investigator is sequentially delivered, and the chaincode is executed. When investigation and identity information, along with crime evidence video transactions, are shared with all of the peers in the two-level blockchain, all of the nodes of each blockchain reach consensus to create a block. At this time, all peers participating in the channel share the same transaction and block. In contrast, if the two-level blockchain is accessed using an unregistered identity, the two-level blockchain system cannot verify the identifier, and the chaincode cannot be modified, as shown in Figure 6. Therefore, the records stored in each ledger cannot be changed, and blocks cannot be created.

## 4. Experimental Results and Analysis

### 4.1. Experimental Environment

The environment and system configuration for testing the performance of the two-level blockchain system are shown in Table 3 and Table 4, respectively, and Hyperledger Caliper was used to test the performance of digital crime evidence video, investigation and identity information, registration, and inquiry. Hyperledger Caliper is one of the Linux Foundation’s Hyperledger benchmarking projects. It is a framework that enables the performance of the blockchain to be tested. Results for a predefined use case can be derived to determine the performance of the blockchain [36].

Hyperledger Caliper’s performance evaluation index supports tests on transaction read throughput, latency, CPU and memory use, and network I/O resource use. Components include the benchmark layer, interface and core layer, and adapter layer. The benchmark layer defines the backend blockchain network and tests arguments to measure performance, while the interface and core layers query the ledger status of the backend blockchain, invoke smart contracts, and generate performance measurement results in HTML format. The adapter layer measures the experiment by interacting with the blockchain and Caliper [37]. To measure the performance of the two-level system, the digital certificates and private keys of each peer and orderer are registered and issued by the authentication server, and the configuration information of the hot and cold blockchains is delivered to the adapter layer.

Next, to deliver multiple transactions to the blockchain and receive responses, a rate controller module is defined in order to deliver transactions at fixed intervals, designated as transactions per second (TPS). In addition, the calling code is implemented so that the experimental module, acting as a real client, can create and deliver transactions. The calling code consists of the *Init*, *Run*, and *End* functions. The *Init* function consists of a blockchain object and context, as well as user-defined arguments, and is called at the beginning of each experiment round. The *Run* function transfers crime evidence videos, investigations, and transactions of identity information to the system, while the *End* function is called at the end of each experiment round to initialize the corresponding function. At this time, all functions in each calling code are processed in an asynchronous manner in order to control response requests and delays occurring between nodes.

### 4.2. Experimental Results

To evaluate the performance of the two-level blockchain system, we evaluated the performance of the storage function and inquiry function of the hot and cold blockchains. To this end, crime evidence videos with capacities of 100 MB to 1 GB and 1 GB to 5 GB, which were selected only in the areas necessary for the facts of presumption, were stored and searched for on the cold blockchain. In the case of crime evidence videos to be stored in the cold blockchain, the capacity was increased in units of 100 MB from 100 MB to 1 GB, and in units of 1 GB from 1 GB to 5 GB, and the blocks were created by repeatedly submitting 100 transactions for each capacity. 

In addition, for the identity and investigation information, for which transactions in the hot blockchain are frequently stored, the amount of data registered when investigating a criminal case in Korea was assumed to be 1 KB. The transmission rate was increased, and 100 transactions were repeatedly submitted in order to create a block. At that point, the TPS was measured by calculating the total number of transactions submitted, the time required, and the transaction processing delay time of the crime evidence videos, as well as identity and investigation information stored in each of the hot and cold blockchains. 

The TPS is the number of transactions that can be processed in one second, and is a representative blockchain performance evaluation index that indicates how many contracts the corresponding blockchain system can process. The transaction processing delay time refers to the delay until a transaction is received and the chaincode is executed. This is used to measure the number of transactions processed per second. Transaction throughput per second calculates the number of transactions processed per second using the first transaction submission time and the last transaction submission time, which is the transaction time, the number of successful and unsuccessful submissions, and the transaction processing delay time. 

Caliper’s rate control module can control the transaction submission rate, number of submissions, and performance calculations. Subsequently, if the experimental module acting as a client repeatedly submits each transaction, including crime evidence videos and identity and investigation information, to the hot and cold blockchains 100 times for 5 rounds, the two-level blockchain system will, upon request, execute the chaincode and create 500 blocks in the configured node. At this time, information such as the number of successful and unsuccessful transaction submissions, submission time, and response time is communicated with the adapter layer in real time during the series of processes from transaction request to block creation. 

**Figure 7:**
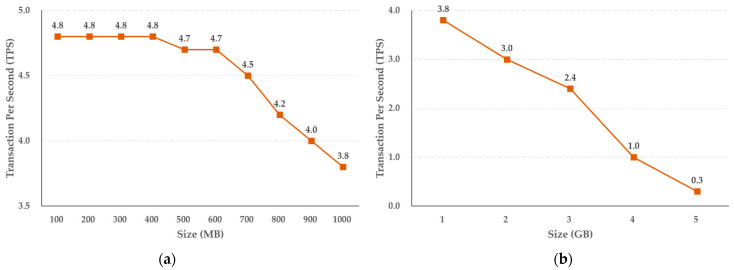
Results of crime evidence video storing performance (**a**) per MB, and (**b**) per GB in the cold blockchain system.

**Figure 8:**
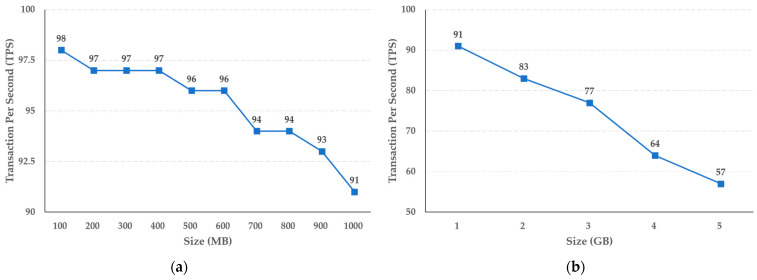
Results of crime evidence video retrieval performance (**a**) per MB, and (**b**) per GB in the cold blockchain system.

The experimental results shown in Figure 7 reveal that the storage function of the cold blockchain decreases by an average of 0.11 TPS when the capacity increases in steps of 100 MB, from 100 MB to 1 GB, while when the capacity increases in steps of 1 GB, the average decrease in TPS is 0.7. In addition, as shown in Figure 8, the query function performance of the cold blockchain decreased by an average of 1.4 TPS when the capacity increased from 100 MB to 1 GB in steps of 100 MB, and by an average of 6.8 TPS when increased in steps of 1 GB. This reflects the difference in the increase in capacity of 100 MB and 1 GB, and confirms that the difference in TPS as the actual uniform capacity increases can be predicted for the target capacity.

**Figure 9:**
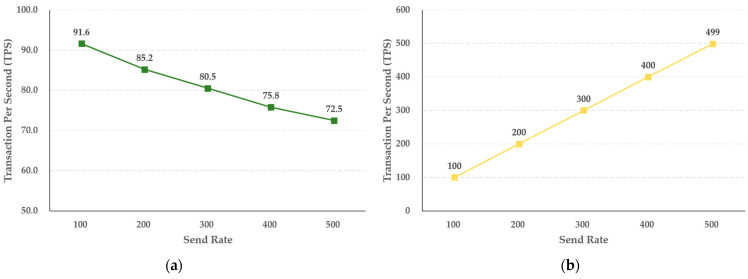
Results of (**a**) saving and (**b**) selecting the performance of identity and investigation information in the hot blockchain by send rate.

According to the experimental results for the hot blockchain, the storage function increases in steps of 100 TPS, from 100 TPS to 500 TPS, as shown in Figure 9, and as the transmission rate increases, the TPS decreases by 3.82 TPS. The inquiry function was also tested in steps of 100 TPS units, from 100 TPS to 500 TPS. As the transfer rate increased, the TPS increased by 79.8 TPS. The query function of the hot and cold blockchains accesses the ledger and state database of the blockchain and only performs a read, so it does not execute a chaincode or create a block. Accordingly, it can be seen that the inquiry function has a higher processing speed than the storage function. In contrast, the storage function of the hot and cold blockchains, which directly generates storage for crime evidence videos and identity and investigation information as blocks in the ledger, is different from the inquiry function, which searches for data stored in the ledger and state database. The storage function of the hot and cold blockchains starts a transaction when a crime evidence video and identity and investigation information are submitted to each blockchain, and if there is no abnormality after verifying the result value of the peer and the digital certificate and private key, the transaction is collected, and blocks are generated. It can be confirmed that the TPS is lower than that of the inquiry function by performing the generation process.

Currently, the typical blockchain systems, Bitcoin and Ethereum, operate at 7 TPS and 20 TPS, respectively, for cryptocurrency transactions for an unspecified number of people. This is numerically superior to the performance of the proposed two-level blockchain system. However, because of the nature of a crime evidence management system, this system can provide similar performance to Bitcoin while storing frequent transactions and large amounts of data separately if the blockchain is accessed by a limited number of users. In addition, because of the technical characteristics of a blockchain, the original crime evidence video can be safely stored and managed inside the blockchain.

## 5. Conclusions and Future Work

The digital crime evidence data obtained during the investigation of a criminal case is transmitted and managed on a physical hard disk until it is analyzed and submitted to the court, and there is a risk that it can be damaged or manipulated by an attack. Therefore, digital crime evidence videos that were difficult to obtain and analyze are not adopted as court evidence, because the continuity of storage cannot be guaranteed. Criminal evidence data are not modified once stored, but investigation information is frequently modified. In this paper, we proposed a two-level blockchain system to increase the integrity of digital crime evidence, so as to efficiently manage criminal evidence. In the proposed system, only authorized participants can access the hot and cold blockchains, in a decentralized environment to separate, store, and share the original information of the investigation, identity information, and digital crime evidence videos. The two-level blockchain system stores the investigation and identity information, as well as digital crime evidence videos, by on-site investigators with verified identities. In addition, by separating data into two blockchains, the same transaction can be stored in the ledgers of all institutions participating in the channel, and the same block can be generated by executing a smart contract. Investigation and identity information, as well as digital crime evidence videos, once created in blocks, cannot be deleted by any user. In addition, because the block is shared with all institutions in the two-level blockchain system, transparency and reliability are enhanced. 

We also evaluated the performance of the proposed system. According to the experimental results, when the storage performance of the cold blockchain increased from 100 MB to 1 GB in units of 100 MB, the average performance decreased by 0.11 TPS. In addition, it was confirmed that when the capacity increased by 1 GB from 1 GB to 5 GB, the decrease was 0.7 TPS on average. The query performance of the cold blockchain had an average decrease of 1.4 TPS when increasing in steps of 100 MB, from 100 MB to 1 GB, and an average decrease of 6.8 TPS when increasing in steps of 1 GB. 

In addition, the experimental results of the hot blockchain revealed that the storage function decreased the TPS by 3.82 when the transmission rate increased from 100 TPS to 500 TPS in units of 100 TPS, and the inquiry performance increased by 79.8 TPS. This is the difference according to the capacity width of the digital crime evidence video used in the experiment, and the transmission rate of the identity and investigation information, and it was confirmed that the actual uniform capacity and the TPS according to the increase in transmission rate were predictable.

The proposed system is suitable for a system that can search while storing data with large files for recording, and ensure the integrity of the data. It can be applied to other applications considering data with similar characteristics, but there is a limitation in applying it to various applications in general. Additionally, for the two-level blockchain system to be used as an actual digital evidence management system, further performance improvements will be required. To this end, as future work, we must first perform an experiment to show the effect of the number of peers in the network. We also intend to research a two-level blockchain system that can respond to high transaction transmission speeds. We also plan to lower the code complexity within the cold blockchain. In the future, it is expected that the proposed two-level blockchain system will contribute to lowering the threat that exists in the transmission and management of digital evidence in Korea, and increasing reliability as a result.

## Figures and Tables

**Table 1:** Amount of digital evidence stored in South Korea as of 2020 [3].

Year	PCs/Laptops	CCTV/EDRs	Smartphones	Databases	Total
2014	3079	510	10,626	654	14,899
2015	3357	712	19,526	700	24,295
2016	3923	794	26,408	1156	32,281
2017	4198	867	30,238	767	36,060
2018	6239	1065	36,986	813	45,103

**Table 2:** Process of the two-level blockchain system.

STEP 1. Register the authentication server ID for identity registration in the two-level blockchain system.
STEP 1-1. The authentication center issues digital certificates and private keys to on-site investigators.
STEP 1-2. Without a digital certificate and private key, the two-level blockchain system cannot be accessed.
STEP 2. Hot and cold blockchain access is granted through a digital certificate and private key.
STEP 2-1. Transactions including investigation and identity information are transmitted to the chaincode of the hot blockchain.
STEP 2-2. Transactions containing a crime evidence video are transmitted to the chaincode of the cold blockchain.
STEP 3. Hot and cold blockchain transactions are saved using digital certificate and private key verification.
STEP 3-1. All judicial institutions in the two-level blockchain system—such as the National Police Agency, the prosecutor’s office, the local police agency, the court, and the cyber analysis team—share transactions.
STEP 3-2. Blocks are distributed to all institutions in the two-level blockchain system through digital certificate and private key validation.

**Table 3:** Experimental environment.

Category	Environmental	Spec. & Version
PC	CPU	AMD Ryzen7 2700 × 3.70 GHZ
	RAM	32 GB
	OS	Ubuntu 16.0 TS
	Storage	Samsung 980 Pro M.2 NVMe SSD 1 TB*2 (RAID-0)
Programming Language	Go	11.4
	Node.js	12.16.3
Framework	Hyperledger Caliper	0.3.1
	Hyperledger Fabric	1.4
	Docker	19.03

**Table 4:** System configuration of the two-level blockchain system for the experiments.

Configuration	Number of Configurations
Peer	5
Orderer	1
State Database	5
Channel	1
Client	5
Certification Authority Server	5
